# Unravelling dynamic trajectories of epistemic emotions in a technology‐enhanced problem‐solving task: A multimodal data approach

**DOI:** 10.1111/bjep.70073

**Published:** 2026-03-16

**Authors:** Tingting Wang, Jianhua Zhang, Shasha Li, Jie Gao, Lingyun Huang, Susanne P. Lajoie

**Affiliations:** ^1^ School of Education Renmin University of China Beijing China; ^2^ Faculty of Education the Chinese University of Hong Kong Hong Kong China; ^3^ Faculty of Education McGill University Montreal Canada; ^4^ Department of Curriculum and Instruction The Education University of Hong Kong Hong Kong China

**Keywords:** emotional trajectories, epistemic emotions, multimodal data

## Abstract

**Background:**

Scholars have reached a consensus on the influential roles and dynamic characteristics of epistemic emotions during the problem‐solving process; however, few research provided empirical evidence to reveal the temporal progression of these emotions.

**Aims:**

This study leverages multimodal data—self‐reports and physiological sensors—to examine variations in a focal epistemic emotion across learning phases and performance levels and to uncover transition patterns among different epistemic emotions.

**Samples:**

A total of 105 students enrolled in a five‐year undergraduate medical program were involved in the data analysis.

**Methods:**

Participants diagnosed a virtual patient in an intelligent tutoring system designed based on the self‐regulated learning (SRL) model. We recorded electrodermal activity (EDA) peaks as an indicator of physiological arousal and collected self‐reports of surprise, curiosity, confusion, enjoyment and boredom across three SRL phases. Two‐way mixed‐design ANOVAs examined variations in the focal emotion and EDA peaks across the forethought, performance and self‐reflection phases, comparing high‐ and low‐performing groups. Temporal network analysis was used to identify transition patterns between emotions during the self‐regulated problem‐solving process.

**Results:**

Epistemic emotions varied distinctly by performance level and SRL phase. For instance, high performers' confusion decreased over time, while low performers showed persistently high confusion. EDA peaks were more frequent during forethought and self‐reflection phases for both groups. More importantly, temporal network analysis revealed several key emotional transitions, including surprise/curiosity → confusion and enjoyment → surprise/boredom.

**Conclusions:**

Overall, this study offers empirical insights into the temporal trajectories of epistemic emotions, informing targeted emotional scaffolding across learning phases.

## INTRODUCTION

Epistemic emotions, such as surprise, curiosity and confusion, constitute a vital subset of academic emotions (Muis et al., 2015; Pekrun et al., 2017) and have been increasingly studied, particularly for their relations to cognitive engagement, self‐regulation strategies and academic outcomes (Chevrier et al., 2019; Vogl et al., 2020; Yang et al., 2024). These emotions are typically defined as those directly associated with the quality of knowledge and knowledge‐generation processes. They often arise when learners encounter discrepancies between new task‐related information and their prior knowledge, beliefs or expectations (Muis et al., 2015; Pekrun et al., 2017). The intensity and nature of these discrepancies give rise to distinct emotional responses: for example, students may feel surprise when faced with unexpected information, which can then develop into curiosity or confusion depending on how manageable the discrepancy is (Muis et al., 2018; Rosman & Mayer, 2018; Vogl et al., 2020). Once the inconsistency is resolved, learners may experience positive emotions such as enjoyment or satisfaction (Muis et al., 2015).

These dynamic shifts indicate that epistemic emotions are not static but fluctuate as learners engage with challenges over time. To better understand these fluctuations, this study adopts Zimmerman's (2000) cyclical phase model of self‐regulated learning (SRL), which divides the learning process into forethought, performance and self‐reflection phases. As students engage in the three SRL phases, they conduct various epistemic activities, such as elaboration and evaluation. This creates an opportunity to observe the emotions students experience and how these emotions change as they transition between different SRL phases. In particular, we explore two temporal dynamics of epistemic emotions: (1) how a specific emotion varies across SRL phases and (2) how transitions occur between different emotions. While theoretical models have proposed such temporal trajectories, e.g., a shift from surprise to confusion, empirical evidence remains scarce. Additionally, further investigation into how emotional trajectories differ between high‐ and low‐performing student groups remains a critical area. By addressing these differences, researchers and developers can design emotion‐aware modules capable of monitoring the temporal fluctuations of epistemic emotions. Based on the emotional trajectories, such systems could then provide tailored and timely interventions to scaffold optimized learning processes.

Moreover, most prior research has relied heavily on self‐reports to measure epistemic emotions, overlooking complementary insights from physiological measures. Electrodermal activity (EDA), which reflects skin conductance changes due to sweat gland activity, has been increasingly used to indicate emotional arousal in learning settings (Boucsein, 2012). For instance, researchers have employed mean EDA levels and the frequency of EDA peaks to infer learners' arousal states (Huber & Bannert, 2023; Nguyen et al., 2023; Zhang et al., 2018). Although EDA excels at capturing dynamic arousal, it cannot distinguish between specific emotional states, highlighting the importance of integrating self‐reports for a fuller understanding of emotional dynamics during SRL. Therefore, this study combines self‐reported data with EDA measures to investigate the temporal dynamics of epistemic emotions across SRL phases in the context of diagnostic problem‐solving.

### Self‐regulated learning in the context of diagnostic problem‐solving

Given the detrimental consequences of misdiagnoses on patient safety and well‐being, it is crucial to promote medical students' diagnostic problem‐solving skills (Dhar et al., 2021; Kulasegaram et al., 2013). Diagnostic problem‐solving is an iterative process whereby medical practitioners gather and analyse patient information, identify physiological abnormalities, order lab tests, formulate hypotheses and reflect on their clinical reasoning (Danielson et al., 2007; Kuiper, 2013). Notably, the complex cognitive demands and high‐stakes nature of diagnostic tasks require learners to engage in self‐regulated learning (SRL) to perform effectively (Lajoie, 2008, 2009; Xu et al., 2023).

SRL refers to a proactive, cyclical process through which learners manage their motivational, emotional, cognitive and behavioural resources to achieve academic goals (Boekaerts 2011; Panadero et al., 2017; Winne & Hadwin, 2008; Zimmerman, 2000).

Scholars in the SRL field have established several prominent theoretical frameworks, such as the COPES model (Winne, 2001), the dual‐processing model (Boekaerts et al., 2000), Pintrich's (2000) model and the metacognitive and affective model (Efklides, 2011). Among the various theoretical models of SRL, this study particularly adopts Zimmerman's (2000) cyclical phase model, which conceptualizes SRL as occurring in three loosely sequential phases: forethought, performance and self‐reflection. The selection of Zimmerman's (2000) model is justified by its advantage in practical significance (Panadero, 2017) and its strong alignment with diagnostic problem‐solving process. Specifically, in the forethought phase, medical students analyse task demands and set goals by identifying critical symptoms and relevant patient information. During the performance phase, they order diagnostic tests, interpret the results, monitor their reasoning and synthesize evidence to propose diagnostic hypotheses. Finally, in the self‐reflection phase, learners evaluate their diagnostic performance, make causal attributions and consider improvements for future clinical reasoning tasks.

### Epistemic emotions and measurements

As medical students engage in various SRL activities, they may experience a range of academic emotions, which can be categorized into achievement emotions, topic emotions, social emotions and epistemic emotions by the object focus (Pekrun & Linnenbrink‐Garcia, 2014). In particular, achievement emotions are directly linked to achievement activities and outcomes, while topic emotions refer to those triggered by the contents covered by learning materials (Pekrun et al., 2011). Social emotions are related to others and typically occur in social interactive situations (Pekrun & Linnenbrink‐Garcia, 2014). Distinct from other academic emotions, the object focus of epistemic emotions is particularly on the quality of knowledge and knowledge‐generation processes (Muis et al., 2018; Pekrun et al., 2017; Pekrun & Loderer, 2020). Specifically, when there is cognitive incongruity, i.e., new information contradicting existing knowledge structures or learners encountering gaps in their understanding, epistemic emotions would be triggered (Pekrun et al., 2017; Vogl et al., 2020, 2021). A substantial body of research has demonstrated the predictive effects of epistemic emotions on cognitive processes, learning strategies and academic performance. For instance, Muis et al. (2015) found that curiosity and enjoyment fostered deeper‐level cognitive processes (e.g., elaboration and critical thinking) and higher learning outcomes, whereas frustration and anxiety were more associated with shallow strategies like rehearsal and thus poorer outcomes. Vogl et al. (2020) further showed that epistemic emotions of surprise, curiosity and confusion tended to trigger diverse knowledge exploration activities. Moreover, the feelings of confusion and frustration hampered students' goal‐setting, planning and metacognitive self‐regulation activities, ultimately leading to disengagement from the learning task (Riegel & Evans, 2021).

Regarding the measurements, self‐report scales, such as the Epistemically‐Related Emotion Scale (EES; Pekrun et al., 2017), were typically used to measure students' retrospective perceptions of epistemic emotions. More recently, the advancement of wearable sensors and multimodal learning analytics enabled a more fine‐grained analysis of physiological signals to indicate emotional experiences. For example, electrodermal activity (EDA), the variations of skin conductivity caused by the changes in sweating of human sweat glands, has been increasingly used to indicate individuals' emotional arousals (Boucsein, 2012; Harley et al., 2015; Strohmaier et al., 2020). Specifically, EDA comprises a slowly‐changing tonic component, skin conductance level (SCL) and a fast‐changing phasic component, skin conductance response (SCR). In educational settings, researchers have extracted the frequency of SCR peaks as an indicator of emotional arousal in various learning contexts, including collaborative learning (Dindar et al., 2020; Järvelä et al., 2023; Törmänen et al., 2023), medical problem‐solving (Harley et al., 2019; Wang et al., 2026) and authentic classroom settings (Yu et al., 2024). Despite the advantage in capturing the temporal dynamics of emotional arousals, EDA lacks the specificity to distinguish between different emotional states, making it essential to combine EDA with self‐report measures to provide a more comprehensive assessment of learners' emotional experiences.

More specifically, sweat gland activity is primarily innervated by the sympathetic nervous system (SNS) (Boucsein, 2012). As individuals experiences various psychological processes, the SNS stimulates the sweat glands and thus alter the skin's conductivity. In this regard, EDA provides a direct, continuous and non‐invasive physiological index to reflect the intensity of affective and cognitive processes, such as the degree of emotional arousals and cognitive load (Boucsein, 2012; Setz et al., 2009). EDA comprises a slowly‐changing tonic component, i.e., skin conductance level (SCL), and a fast‐changing phasic component, i.e., skin conductance response (SCR).

### The dynamic evolutions of epistemic emotions during SRL

The relationship between SRL and epistemic emotions is inherently reciprocal. On the one hand, epistemic emotions are elicited by the progression of SRL activities (Muis et al., 2018). During the forethought phase, students' analyses of the complexity and novelty of learning materials and their perceptions of control and task value serve as primary triggers for specific emotional states (Muis et al., 2018). As students transition into the performance phase, they deploy cognitive and metacognitive strategies to navigate unfamiliar or conflicting information; this process often facilitates the evolution of epistemic emotions, such as the shift from curiosity to confusion (Efklides & Schwartz, 2024). Finally, in the self‐reflection phase, the cognitive conditions established through self‐evaluation and metacognitive attributions further influence emotional transitions.

On the other hand, epistemic emotions function as internal feedback mechanisms that inform subsequent SRL processes, including goal‐setting, planning and strategy selection (D'Mello & Graesser, 2012). For instance, experiencing confusion during task definition may diminish a learner's self‐efficacy, prompting them to set more attainable goals. In contrast, the presence of curiosity during task analysis often correlates with the pursuit of more ambitious learning objectives. Ultimately, epistemic emotions are deeply embedded within the temporal unfolding of SRL, necessitating a closer examination of how these affective states evolve alongside regulatory processes.

Researchers have proposed several theoretical assumptions to describe the developmental trajectories of these emotions. Typically, when learners encounter novel information that contradicts their prior knowledge or beliefs—referred to as cognitive incongruity—surprise emerges as the initial emotional response (D'Mello & Graesser, 2014; Theobald et al., 2021). This surprise may then transition into curiosity, confusion or both, depending on how learners appraise the complexity of the information, their perceived competence and the task's value (Muis et al., 2018). Specifically, manageable complexity and high task value tend to foster curiosity (Silvia, 2010), while excessive complexity or low perceived competence may elicit confusion (Di Leo et al., 2019). As learners continue, curiosity can lead to enjoyment or delight if their epistemic goals are achieved. Conversely, persistent confusion—especially when paired with a declining sense of control—may escalate into anxiety, frustration or boredom (Pekrun et al., 2010). Nevertheless, successful resolution of confusion can also result in enjoyment or satisfaction (Berweger et al., 2023; Bosch & D'Mello, 2017; Radoff et al., 2019; Vilhunen et al., 2023).

Despite these well‐established theoretical perspectives, empirical evidence on the temporal dynamics of epistemic emotions remains limited. To our best knowledge, a small body of empirical studies explored transitions of epistemic emotions within the SRL cycle. Di Leo et al. (2019) identified students' epistemic emotions using think‐aloud protocols and reported several statistically significant emotional transitions, such as confusion → confusion, surprise → confusion and confusion → boredom. Zheng et al. (2023) extended Di Leo et al.'s work into a phase‐specific SRL framework. They tracked academic emotions across three SRL phases: forethought, performance and self‐reflection. Using self‐report measures and growth modelling, they found that curiosity and confusion declined across phases, while boredom increased in the self‐reflection phase. Additionally, curiosity and enjoyment in the initial phase were found to positively predict students' performance. However, how students' epistemic emotions transitioned across the unfolding SRL phases and how these phase‐specific patterns differ among students with varying performance levels remain underexplored.

Therefore, the present study aims to employ a multimodal learning analytics approach, involving physiological features and a temporal network analysis, to examine students' dynamic epistemic emotions as they accomplish a self‐regulated diagnostic problem‐solving task. Given the limitation in related empirical results, this study cannot propose specific directional hypotheses but remains exploratory in nature. Specifically, this study aims to address the following three research questions (RQs):

*How did epistemic emotions differ across distinct performance levels and SRL phases?*

*How did discrete epistemic emotions evolve over three SRL phases?*

*Which emotions, within the identified emotional trajectories, demonstrated the strongest dependencies on other emotions?*

## METHODS AND MATERIALS

### Participants

A total of 112 medical students from a large research‐intensive university in the northeastern Chinese Mainland volunteered to participate in this study. All participants were in the fourth year of a five‐year undergraduate medical program. Since this study employed the physiological signal to indicate emotional arousals, seven participants were excluded due to low‐quality signal, leading to a sample of 105 students included in the final data analysis. The sample consisted of 63 female and 42 male students, with an average age of 22.70, SD = 2.54. Prior to data collection, we had obtained the approval of a Research Ethics Board. Moreover, participants had completed several prerequisite courses, such as endocrinology, metabolism and pathology, ensuring that they mastered the prior knowledge required for solving the diagnostic problem‐solving tasks involved in this study.

### Learning contexts and tasks

**FIGURE 1:**
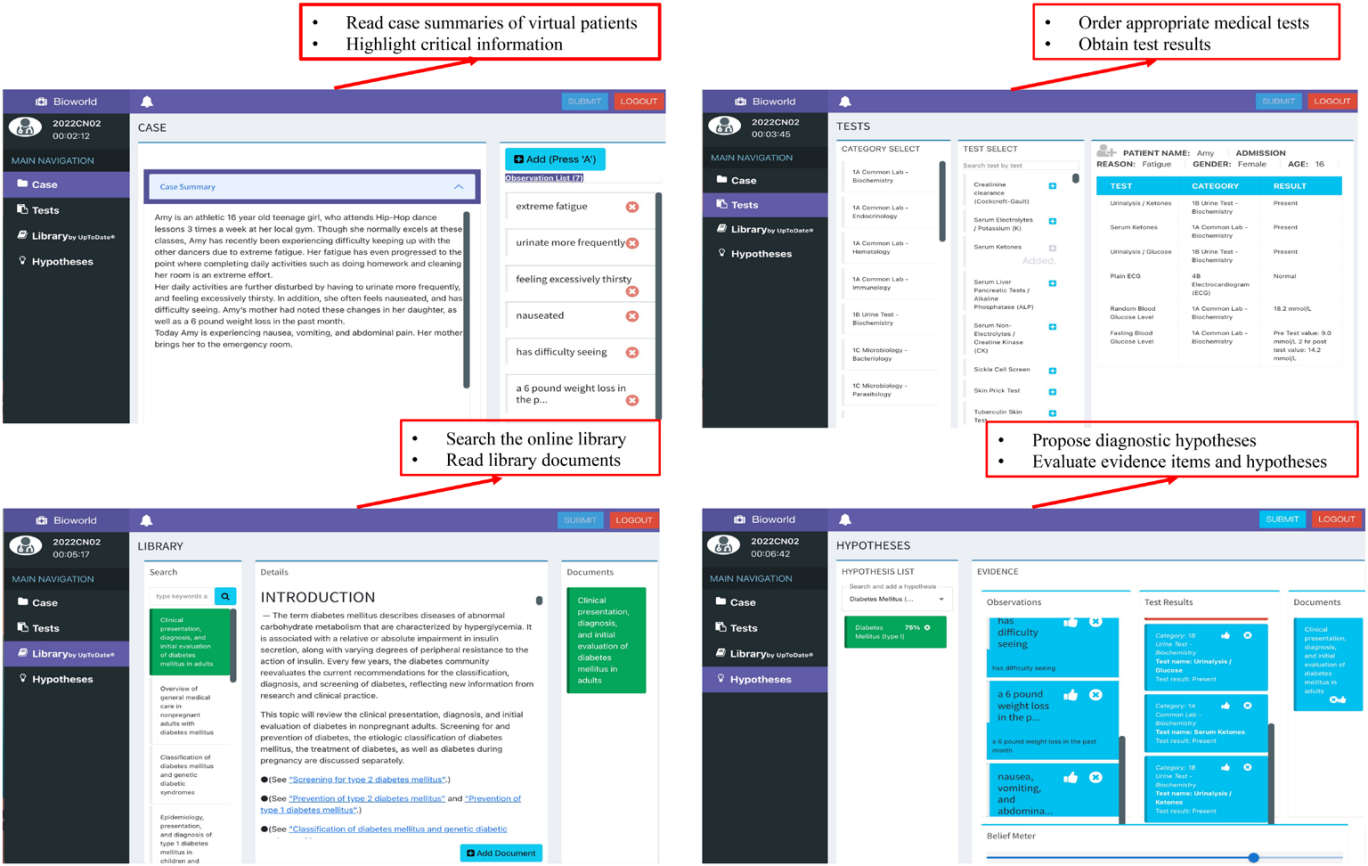
The BioWorld interface.

Participants completed a diagnostic problem‐solving task in the context of BioWorld (see Figure 1), an intelligent tutoring system simulating virtual patients to cultivate medical students' diagnostic expertise. Designed based on the principles of SRL frameworks, BioWorld supported medical students' self‐regulated problem‐solving processes (Lajoie, 2009, 2021). Specifically, the system enables participants to analyse virtual patients (i.e., *forethought*), conduct diagnoses (i.e., *performance*), and evaluate and reflect on their problem‐solving processes (i.e., *self‐reflection*). During the *forethought phase*, medical students were guided to conduct task analysis by reviewing a case summary describing basic information and symptoms of the virtual patient. As well, they identified and highlighted the critical evidence from the case summary, which would be gathered into an observation list for further review. During the *performance* phase, BioWorld afforded students to collect more information by ordering medical tests, collecting lab test results and searching an online library to formulate one or more diagnostic hypotheses. Moreover, they were scaffolded to monitor their task progress by evaluating the supportiveness of collected evidence and assess the possibility of proposed hypotheses. Students were required to submit a final decision to end the *performance* phase. During the *self‐reflection* phase, medical students wrote a reflective summary to elaborate on how they made the final diagnostic decision.

### Procedures

**FIGURE 2:**
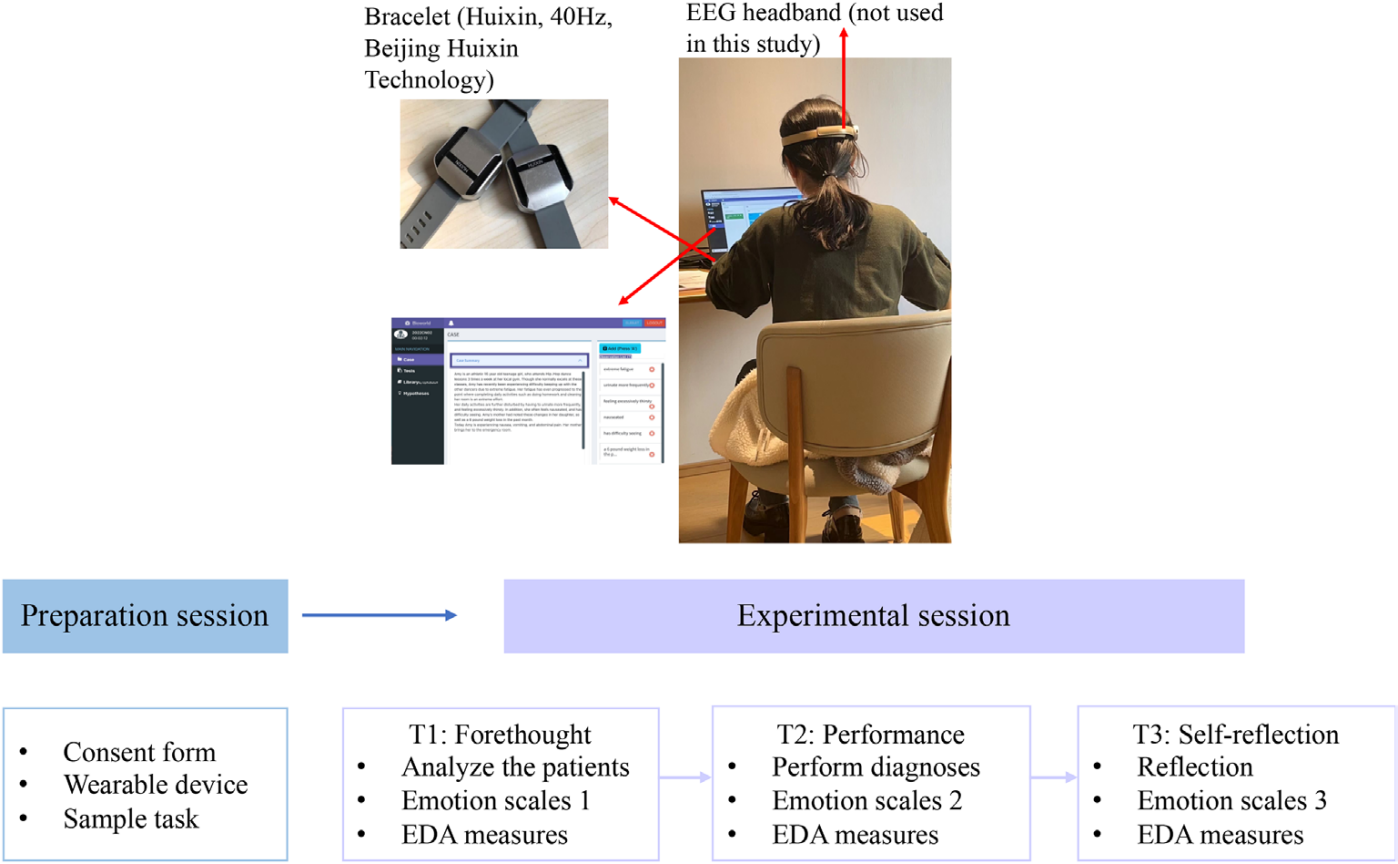
Research procedures.

As shown in Figure 2, this study comprised a preparation and an experimental session. During the preparation stage, students reviewed and signed the consent form and were then equipped with a psychophysiological device to record their EDA in real time. Afterwards, they were provided with a sample task to familiarize themselves with the BioWorld system. In the experimental session, students diagnosed the virtual patient Cynthia and self‐reported their epistemic emotions at three points: after analysing patient information and before any active operations (i.e., *forethought*), after submitting the final diagnosis (i.e., *performance*) and after reviewing the individualized feedback (i.e., *self‐reflection*). Moreover, their EDA was captured throughout the entire diagnostic problem‐solving process.

### Measures

#### Task performance

In the present study, students were tasked with diagnosing a virtual patient Amy, who presented with Type I Diabetes. Task performance was operationalized as diagnostic accuracy, a binary variable indicating whether the participant arrived at the correct clinical diagnosis. Within the BioWorld environment, performance feedback was automatically generated upon the completion of all prescribed learning activities. Participants were subsequently categorized into high‐performing and low‐performing groups based on their diagnostic success or failure, respectively.

#### Discrete epistemic emotions

This study employed the Epistemically‐Related Emotion Scale (EES) developed by Pekrun et al. (2017) to measure students' surprise, confusion, curiosity, enjoyment and boredom during three SRL phases. Each emotion was assessed by three items ranging from 1 (not at all) to 5 (very strong) and each item consisted of a single adjective describing the emotion. For instance, the words “curious,” “inquisitive,” and “interested” were used to measure curiosity. The Cronbach's alpha values were all above .70, suggesting satisfactory internal consistency.

#### EDA recording and pre‐processing

To record EDA signals, researchers instructed participants to wear a psychophysiological sensor (Psychorus, Huixin, China) on their left wrist, as all participants were all right‐handed (see Figure 2). This device has been used to capture students' EDA in a variety of authentic learning settings (Yu et al., 2024; Zhang et al., 2018). This sensor records EDA at a sampling rate of 40 Hz using four surface electrodes with conductive gels.

**FIGURE 3:**
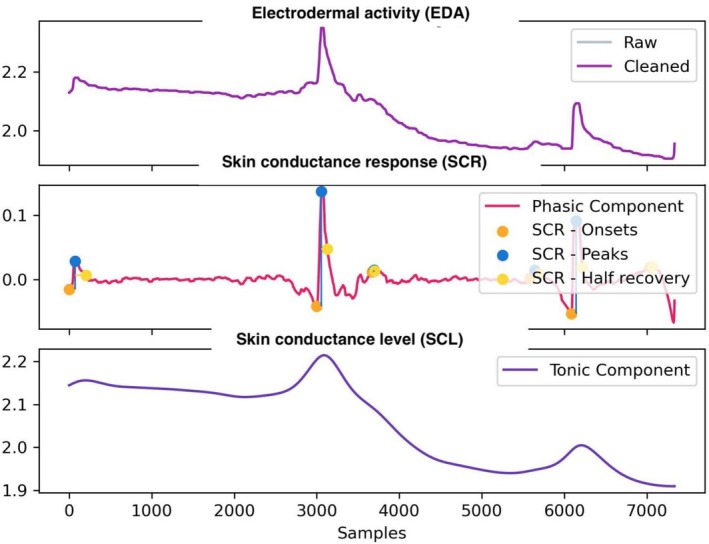
An example of EDA signals. The unit of EDA is ms, and the x‐axis refers to the number of data points. Since the EDA signal has a sampling rate of 40 Hz, which indicates that there will be 40 data points per second. The upper panel reflects the raw and cleaned EDA signals. Moreover, it is worth mentioning that there were minor visual differences between the raw and cleaned EDA signals. The middle panel represents the fast‐changing SCR component, with the blue dots indicating SCR peaks. The lower panel demonstrates the slowly changing SCL components.

Before conducting statistical analyses, we first aligned the EDA signals with the time stamps of the three SRL phases. During students' diagnostic problem‐solving, their interactive operations within BioWorld as well as corresponding timestamps were automatically recorded in log files. We extracted the time periods for the *forethought*, *performance* and *self‐reflection* phases of SRL and segmented EDA signals according to these timestamps. Then human inspections were conducted to exclude low‐quality EDA signals based on the proportion (i.e., ≤ 20%) of artefacts, leading to an inclusion of only 105 participants in the final data analysis. After that, we performed a low‐pass filter and a 4th‐order Butterworth filter to further remove signal noises, using a Python package “NeuroKit 2” (Makowski et al., 2021). EDA was then decomposed into SCL and SCR components, and the frequency of SCR peaks were extracted for further data analyses (see Figure 3). SCR_peaks indicate the number of peaks per minute over a specific SRL phase, considering each SRL phase lasted variedly.

### Data analysis

To address RQ1, we conducted two‐way mixed‐design ANOVAs and Bonferroni‐corrected post‐hoc analyses to explore the variations of each epistemic emotion and SCR_peaks across three SRL phases (within‐subject factor), considering the effects of performance levels (between‐subject factor). Specifically, this study examined both the main and interaction effects of performance level and SRL phase.

To reveal the temporal evolutions of epistemic emotions (RQ2), a psychometric network analysis was performed. To be specific, the *temporal network* was estimated to examine how a variable at time *t* − 1 predicts variables at time *t*, when accounting for all other variables at time *t* − 1 (Beymer et al., 2022; Malmberg et al., 2022; Saqr, 2024; Xiang et al., 2024). Given that epistemic emotions in this study were measured at only three time points, the stationarity can be assumed for the variables over this short period. Significant temporal edges in the *temporal network* can indicate the evolution of emotions across the three SRL phases. The *temporal network* also estimated the autoregressive effects, manifested by significant self‐loops. For network estimations, this study performed three steps to select the best‐fitting model: First, a saturated model including all edges was estimated. Then we performed a pruned model to include only significant edges (*p* < .05). The last step used the *stepup* function to add the edges with the strongest modification index stepwise until the Bayesian information criterion (BIC) of the model no longer improves (Epskamp, 2020, 2021). The optimal model was considered as the one with a lower Akaike information criterion (AIC) and BIC value. A chi‐square difference test was also performed to compare the models.

To address the RQ3, we computed the strength centrality to identify which variables are more influential in the networks. The computation of strength of centrality in *temporal networks* is divided into in‐strength and out‐strength. In‐strength centrality is the sum of absolute values of all significant incoming edges, while out‐strength centrality is the sum of absolute values of all significant outgoing edges (Freeman, 1978; Opsahl et al., 2010). Variables with high in‐strength and out‐strength centrality suggest that they are well predicted by other variables and that they can strongly predict other variables in the network, respectively. In this study, we standardized the strength indices for further comparisons.

## RESULTS

### RQ1. How did students' epistemic emotions differ across distinct performance levels and SRL phases?

**FIGURE 4:**
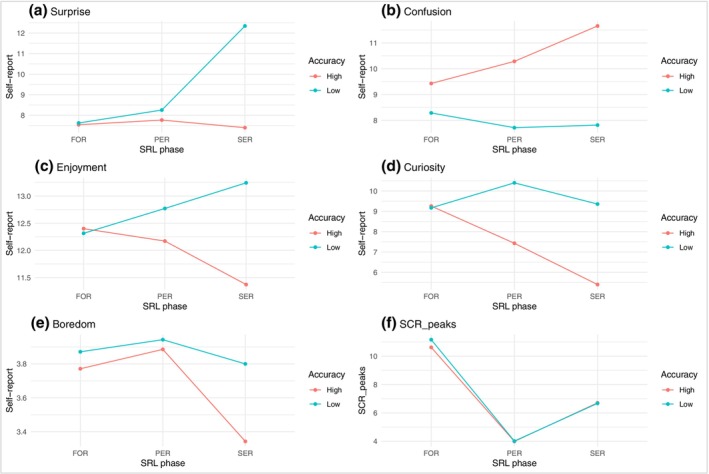
Post‐hoc analysis result.

Table 1 first provides the descriptive statistics of self‐reported epistemic emotions and physiological arousals. Table 2 summarizes the effects of performance level (high vs. low) and SRL phases on epistemic emotions, with post‐hoc results in Figure 4. Specifically, significant main effects of performance level (*F* = 8.84, *p* = .004) and SRL phase (*F* = 31.36, *p* < .001), as well as their interaction (*F* = 38.61, *p* < .001), were found for surprise. Post‐hoc analyses (Figure 4a) revealed that high performers' surprise remained stable across SRL phases, whereas low performers experienced greater surprise in the self‐reflection phase (*M* = 12.34) compared to the forethought (*M* = 7.63) and performance phases (*M* = 8.26), *t* = 12.12, *p* < .001, and *t* = 11.83, *p* < .001. (The readers can find more detailed information in Appendix A: Table A1).

**TABLE 1:** Descriptive statistics of self‐reported epistemic emotions and physiological arousals.

Group	SRL	Surprise		Confusion		Enjoyment	
		*M*	SD	*M*	SD	*M*	SD
High Performers	FOR	7.54	3.27	9.26	3.49	9.43	3.52
	PER	7.77	3.73	7.43	3.67	10.29	3.42
	SER	7.40	3.45	5.40	2.77	11.66	3.11
Low Performers	FOR	7.63	3.38	9.17	3.54	8.29	3.19
	PER	8.26	3.52	10.40	3.69	7.71	3.41
	SER	12.34	3.36	9.36	3.60	7.81	3.35

Group	SRL	Curiosity		Boredom		SCR_peaks	
		*M*	SD	*M*	SD	*M*	SD
High Performers	FOR	12.40	2.49	3.77	12.40	2.49	3.77
	PER	12.20	2.70	3.89	12.20	2.70	3.89
	SER	11.40	2.94	3.34	11.40	2.94	3.34
Low Performers	FOR	12.30	2.32	3.87	12.30	2.32	3.87
	PER	12.80	2.24	3.94	12.80	2.24	3.94
	SER	13.20	2.61	3.80	13.20	2.61	3.80

Abbreviations: FOR, forethought; PER, performance; SER, self‐reflection.

**TABLE 2:** Effects of performance levels and SRL phases on epistemic emotions.

Term	Surprise *F*	Confusion *F*	Enjoyment *F*	Curiosity *F*	Boredom *F*	SCR_Peaks *F*
Performance	8.84**	15.04***	18.97***	3.32	.50	.03
SRL phase	31.36***	13.91***	4.22*	0.24	2.17	23.18***
Interaction	38.61***	15.94***	8.66***	8.37***	.83	.05

*Note*: Greenhouse–Geisser corrections were used due to sphericity violations. *p < .05, **p < .01, ***p < .001.

Similarly, the results also showed that performance level and SRL phase had significant main and interaction effects on confusion (*F* = 15.04, *p* < .001; *F* = 13.91, *p* < .001; *F* = 15.94, *p* < .001) and enjoyment (*F* = 18.97, *p* < .001; *F* = 4.22, *p* = .016; *F* = 8.66, *p* < .001). As illustrated by Figure 4b, post‐hoc analyses indicated that high performers reported the most confusion in the forethought phase (*M* = 9.26) compared to the performance (*M* = 7.43) and self‐reflection phases (*M* = 5.40), *t* = 3.18, *p* = .006, and *t* = 3.86, *p* < .001. Conversely, low performers experienced higher confusion during the performance phase (*M* = 10.40) than forethought (*M* = 9.17), *t* = 3.02, *p* = .010.

Regarding the emotion of enjoyment (Figure 4c), it varied with SRL phases only for high performers, who experienced greater enjoyment in self‐reflection (*M* = 11.66) than forethought (*M* = 9.43) and performance phases (*M* = 10.29), *t* = 4.02, *p* < .001. In contrast, low performers exhibited nonsignificant differences in the feelings of enjoyment over the three SRL phases.

Furthermore, the results also suggested that performance level and SRL phase had no significant main effects on curiosity and boredom, though curiosity showed a significant interaction, *F* = 8.37, *p* < .001. As shown in Figure 4d, post‐hoc analyses indicated that low performers were more curious during the self‐reflection phase (*M* = 13.20) compared to the forethought phase (*M* = 12.30), *t* = 2.76, *p* = .021, while high‐performing students were not statistically distinct in curiosity across three SRL phases. In terms of boredom, both high and low performers reported a relatively low‐level boredom, without significant variations during three SRL phases.

Additionally, we analysed how students with different performance experienced physiological arousals across the forethought, performance and self‐reflection phase of SRL. The results indicated nonsignificant differences in SCR_peaks between high‐ and low‐performing students; however, SRL phases significantly influenced physiological arousals, *F* = 23.18, *p* < .001. Specifically, the frequency of peaks per minute (SCR_peaks) was highest in the forethought phase (*M* = 10.89), followed by self‐reflection (*M* = 6.69), *p* = .003 and performance phase (*M* = 4.00), *p* < .001.

### RQ2. How did discrete epistemic emotions evolve over three SRL phases?

As aforementioned, to select the best‐fitting model, we compared the saturated, pruned and step‐up models according to BIC, AIC and the chi‐squared difference test results. The fit indices for the three models were as follows: saturated: BIC = 111,631.20, AIC = 11,406.05; pruned: BIC = 11,666.22, AIC = 11,564.90; stepup: BIC = 11,544.87, AIC = 11,413.53. Notable increases in both AIC and BIC values were observed from the saturated model to the pruned model, while the values of BIC declined and AIC increased from the saturated model to the stepup model. Additionally, the chi‐square difference test between the saturated and stepup models yielded a significant result, χ^2^ (25) = 57.48, *p* < .001, indicating that the stepup model significantly worsened the fit compared to the saturated model. Based on these indices, we selected the saturated model in this study as the *temporal network*.

**FIGURE 5:**
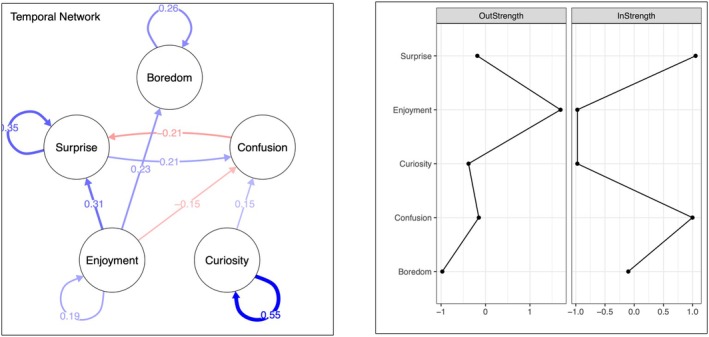
The temporal network of self‐reported epistemic emotions (left) and centrality indices (right). The edges and self‐loops depicted in the left panel were all statistically significant (*p* < .05). The centrality indices in the right panel were all standardized.

Figure 5 (the left panel) and Table 3 depict the temporal network of five epistemic emotions, with blue and red edges reflecting significantly positive and negative predictive effects of emotions at time *t‐1* on those at time *t*, respectively. Specifically, the self‐loops revealed substantial autoregressive effects for surprise, curiosity, enjoyment and boredom during the SRL process, while the autoregression of confusion was nonsignificant. Moreover, the network model exhibited temporal dependencies of distinct emotions. The edge surprise‐ > confusion (*Est* = .21, *p* = .005) showed that students' feelings of surprise during a previous SRL stage could positively predict their experienced confusion at a later stage. Similarly, we also found curiosity was a positive predictor of subsequent confusion (curiosity‐ > confusion; *Est* = .15, *p* = .031). Furthermore, enjoyment had a positive forward influence on surprise (enjoyment‐ > surprise; *Est* = .31, *p* < .001) and boredom (enjoyment‐ > boredom; *Est* = .23, *p* < .001) and a negative forward influence on confusion (enjoyment‐ > confusion; *Est* = −.15, *p* = .018). Additionally, there was another negative edge from confusion to surprise (confusion‐ > surprise; *Est* = −.21, *p* = .004), suggesting more intense confusion in a previous SRL phase tended to predict less intense surprise in the following phase.

**TABLE 3:** Temporal relations between epistemic emotions.

Predictors (t − 1)	Outcomes				
	Surprise	Curiosity	Confusion	Enjoyment	Boredom
Surprise	.35*** (.08)	.03 (.05)	.21** (.07)	.12 (.09)	.03 (.07)
Curiosity	−.03 (.05)	.55*** (.07)	.15* (.07)	.13 (.08)	.02 (.07)
Confusion	−.21** (.07)	.11 (.08)	.13 (.08)	.05 (.08)	−.07 (.07)
Enjoyment	.31*** (.06)	−.01 (.07)	−.15* (.06)	.19* (.09)	.23*** (.06)
Boredom	−.10 (.07)	−.03 (.08)	.13 (.07)	.15 (.09)	0.26** (.09)

*Note*: The estimates indicate the temporal effects of rows on columns. *p < .05, **p < .01, ***p < .001.

### RQ3. Which emotions demonstrated the strongest dependencies on other emotions?

To address this question, the *in‐strength* and *out‐strength centrality* were used to reflect the relative importance or impacts of five epistemic emotions in the temporal network model. Moreover, the values of centrality indices were standardized for direct comparisons. As illustrated by the right panel of Figure 5, we found that enjoyment had the highest *out‐strength*, followed by confusion, surprise and curiosity, with boredom having the lowest *out‐strength*. These findings suggested that enjoyment during the previous SRL phase was a key predictor of other emotional states in the following phase. In contrast, surprise and confusion had the highest *in‐strength centrality* and were most strongly predicted by other emotions experienced during previous SRL phases.

## DISCUSSION

This study examined the temporal dynamics of epistemic emotions, measured by self‐report scales and physiological sensors, across three SRL phases while students engaged in a diagnostic problem‐solving task within BioWorld. In particular, we investigated both the temporal variations of a focal epistemic emotion and the evolving transitions among five distinct epistemic emotions. Specifically, a series of two‐way mixed‐design ANOVAs were conducted to examine how specific epistemic emotions and emotional arousals fluctuated across the three SRL phases, and how these patterns differed between high and low performers. The finding indicated that high‐performing students exhibited stable levels of surprise throughout the task, while their feelings of confusion significantly declined and enjoyment progressively increased across the forethought, performance and self‐reflection phases. One possible explanation is that high‐performing students may have encountered substantial unfamiliar or conflicting information during the forethought phase (Muis et al., 2015, 2018), particularly while analysing the virtual patient, which triggered heightened confusion. As they actively engaged with the task and applied effective learning strategies to resolve the discrepancies, their confusion diminished and positive emotions such as enjoyment became more prominent in the subsequent phases. In contrast, low‐performing received negative performance feedback beyond their expectations during the self‐reflection phase, leading them to experience the greatest surprise and curiosity at this stage. As well, those low‐performing individuals were required to conduct various medical tests and integrate tests results and other information to solve the diagnostic task and thus experienced more intense confusion during the performance phase. Distinct from high‐performing students, low‐performing students were less likely to generate enjoyment over the whole problem‐solving task and did not demonstrate significant differences in across the three SRL phases. Moreover, both high‐ and low‐performing medical students actively engaged in the problem‐solving task and did not feel high‐level boredom.

In addition to the intensity of specific epistemic emotions, this study also captured students' EDA signals to indicate their overall physiological arousals during three SRL phases. EDA, an objective measure of dynamic emotional arousals, has been growing used in a range of educational settings (Yu et al., 2024; Zhang et al., 2018). In this study, we particularly analysed the fast‐changing component (i.e., SCR) and extracted the indicator, SCR_peaks, which was computed as the number of SCR peaks per minute (Harley et al., 2019; Wang et al., 2026; Zheng et al., 2024). Surprisingly, we did not observe significant differences in SCR_peaks between high‐ and low‐performing students. In contrast, as previously noted, these two groups demonstrated significantly different subjective experiences of surprise, confusion and enjoyment. These findings indicate that uniform physiological data may obscure underlying variations in subjective affective states, highlighting the necessity of integrating multiple data modalities to more accurately capture the multidimensional nature of epistemic emotions. However, the results showed that SRL phases mattered in affecting the variations of SCR_peaks. Specifically, students' emotional arousals were found to be the highest during the forethought phase, followed by the self‐reflection and performance phases. This finding indicated that the preparation activities during the forethought phase, such as identifying task difficulty and goal‐setting, evoked heightened emotional responses. As students enacted the task by conducting various cognitive and metacognitive strategies during the performance phase, they gradually calmed down and experienced less intense emotions. However, reflective summary and feedback could also evoke their emotional responses in the self‐reflection phase.

Although it is important to examine the temporal dynamics of specific epistemic emotions, it is worth more investigations of the interrelatedness of different emotions from a perspective of complex dynamic system (Li et al., 2022). In particular, leveraging dynamic network analysis, this study uncovered the transitional patterns of epistemic emotions across three SRL phases. Interestingly, the results indicated that enjoyment served as a strong predictor of other emotions, while surprise and confusion were more often predicted by preceding emotional states. Regarding the specific trajectories, the findings showed that all epistemic emotions except confusion exhibited significant autoregressive effects, reflecting their persistence and continuity over a relatively short learning period. The missing self‐loop suggested that students' feelings of confusion was not sustained and also reflected students' active engagement in resolving cognitive discrepancies (Beymer et al., 2022; Malmberg et al., 2022).

More importantly, significant predictive effects were observed between pairs of different epistemic emotions, which suggested interconnected trajectories and dynamic transitions between these emotions. Firstly, consistent with the theoretical assumption of the “surprise–curiosity–confusion” relationship (Vogl et al., 2020), we found that confusion in a later SRL phase was positively predicted by prior experiences of surprise and curiosity. In this study, surprise could be triggered by unexpected patient information, symptoms and lab test results that contradicted with or exceeded medical students' current expertise. The emotional state of surprise would evolve into confusion when they perceived the discrepant information as too challenging and impossible to resolve. As well, the trajectory from curiosity to confusion also indicated the discrepancies were incomprehensible. Secondly, the strongest predictive effects of enjoyment on other emotions, e.g., surprise and boredom, were striking. The transition from enjoyment to surprise could be explained by the fact that our participants were not hedonistic but sustained information processes to acquire more knowledge and develop diagnostic expertise. In contrast, the evolutionary trajectory from enjoyment to boredom might suggest students' success in the problem‐solving task and a fade of engagement after resolving the discrepant information (Zhu et al., 2024). Thirdly, there were also two significantly negative edges (in red). When students experienced intense enjoyment at a previous SRL stage, they were less likely to feel confused in subsequent SRL stages. This negative relationship was reasonable, as enjoyment arose when students resolved the discrepant information or confirmed their hypotheses (Muis et al., 2018), indicating a smooth problem‐solving process and a low likelihood of confusion. The negative associations between confusion to surprise might suggest that confusion could not evolve into surprise.

### Practical implications

Overall, this study demonstrates that emotional trajectories are phase‐sensitive and performance‐contingent, which has important implications for the design of SRL scaffolds and technology‐based learning environments. Specifically, our findings can inform which emotions should be monitored or prompted at different SRL phases to optimize learning. For instance, the low level of enjoyment across SRL phases observed in low‐performing students suggests that tasks should be designed to provide incremental successes. This could be achieved through adaptive, stepwise hints that activate prior knowledge or guide learners towards the next actionable step.

Meanwhile, a systematic decline in confusion among high‐performing students suggests that the absence of such a decline in other learners necessitates timely interventions. Upon detecting prolonged confusion, the system interface should provide immediate scaffolds, such as more elaborated information, guiding questions or inquiry prompts, to help learners resolve discrepant information and prevent unresolved confusion. Additionally, the observed decoupling between physiological arousal and subjective emotional experience across high‐ and low‐performing students highlights the importance of using multimodal indicators to understand SRL. These findings suggest that physiological signals may simply reflect general arousals (Romine et al., 2022), rather than a specific emotional state. Thus, from a pedagogical standpoint, the design of instructional technologies requires the integration of both subjective and objective data sources to help dynamically capture emotional variations as well as clarify the qualitative meaning of these changes.

### Limitations

Despite these remarkable findings, this study is not without limitations. First, participants reported their perceived epistemic emotions at only three time points to minimize interruptions to their cognitive processes. Future research could benefit from employing think‐aloud protocols and facial expression analyses, which allow for the observation of learners' fine‐grained emotional responses. Second, due to the relatively small sample size, this study did not compare the temporal networks of epistemic emotions between high‐ and low‐performing students to maintain an acceptable model fit. Future investigations should consider examining these differences, as exploring emotional transitions across performance levels may yield valuable insights. As well, individual factors, such as trait emotions and prior knowledge, matter in interpreting diverse emotional trajectories, requiring the incorporation of these factors into future research. Third, this study was conducted in the context of a diagnostic problem‐solving task within an intelligent tutoring system. Given that different technologies may affect learners' cognitive and affective processes in distinct ways, future research can explore the dynamics of epistemic emotions in interactions with generative artificial intelligence tools. Another notable limitation of the current study is the use of a virtual learning environment BioWorld. While the simulated virtual patients enable a safe training environment for medical students, the low‐stakes nature may lead to diminished emotional involvement compared to real‐world clinical settings. In actual practice, the ethical and professional responsibility for a patient's health likely amplifies the intensity of epistemic emotions like confusion. Future research should be conducted in high‐ecology situations to reveal the emotional trajectories in authentic problem‐solving tasks.

## CONCLUSION

In conclusion, this study employed multimodal data and temporal network analysis to examine the dynamic trajectories of epistemic emotions across the forethought, performance and self‐reflection phases of SRL. The findings revealed that high‐performing students experienced significant variations in confusion and enjoyment across SRL phases, whereas low‐performing students demonstrated notable shifts in surprise, confusion and curiosity. Additionally, both groups exhibited heightened physiological arousals during the forethought phase than the self‐reflection and performance phases. With respect to the temporal transitions of epistemic emotions, this study provided empirical support for theoretical assumptions—such as the progression from surprise and curiosity to confusion—and uncovered additional patterns, including transitions from enjoyment to surprise and boredom. These findings contribute theoretically by validating conceptual models and offering a more nuanced understanding of the interplay between epistemic emotions and SRL processes. Methodologically, the integration of electrodermal activity (EDA) signals and temporal network analysis illustrates the potential of multimodal learning analytics to advance research on learners' cognitive and affective processes. From a practical perspective, the findings offer valuable insights for educators and technology designers to monitor and respond to students' emotional trajectories. For example, a transition from surprise to confusion may signal excessive task difficulty, suggesting the need for conceptual, strategic or metacognitive scaffolds to support learners effectively. The progression from enjoyment to boredom indicates disengagement from learning, which necessitates immediate prompts from instructors or educational technologies to help learners sustain active engagement in learning tasks. Taken together, these insights underscore the importance of designing adaptive learning environments and instructional scaffolds that are sensitive to learners' emotional dynamics across different SRL phases, ultimately fostering more personalized and effective educational experiences.

## AUTHOR CONTRIBUTIONS

**Tingting Wang:** Conceptualization; methodology; formal analysis; writing – review and editing; writing – original draft; funding acquisition; data curation. **Jianhua Zhang:** Methodology; formal analysis; writing – original draft. **Shasha Li:** Writing – original draft. **Jie Gao:** Writing – original draft; investigation. **Lingyun Huang:** Writing – review and editing; validation. **Susanne P. Lajoie:** Writing – review and editing.

## CONFLICT OF INTEREST STATEMENT

The authors declare that they have no competing interests.

## ETHICS APPROVAL

Ethical standards were followed in the conduct of the study, including Institutional Review Board approval for all research activities and consent of all participants.

## Data Availability

The datasets generated during and/or analysed during the current study are available from the corresponding author.

## APPENDIX A

**TABLE A1:** The detailed indicators of two‐way mixed‐design ANOVA.

Outcome	Term	*F*	df1	df2	MSe	*η* ^*2*^_*p*_
Surprise	Performance	8.84**	1.00	103.00	12.40	0.08
	SRL Phase	31.36***	1.64	168.92	8.50	0.23
	Interaction	38.61***	1.64	168.92	8.50	0.27
Confusion	Performance	15.04***	1.00	103.00	10.15	0.13
	SRL Phase	13.91***	1.82	187.46	7.20	0.12
	Interaction	15.94***	1.82	187.46	7.20	0.13
Enjoyment	Performance	18.97***	1.00	103.00	11.20	0.16
	SRL Phase	4.22*	1.76	181.28	8.10	0.04
	Interaction	8.66***	1.76	181.28	8.10	0.08
Curiosity	Performance	3.32	1.00	103.00	13.50	0.03
	SRL Phase	0.24	1.90	195.70	9.80	0.00
	Interaction	8.37***	1.90	195.70	9.80	0.08
Boredom	Performance	0.5	1.00	103.00	14.20	0.01
	SRL Phase	2.17	1.80	185.40	10.50	0.02
	Interaction	0.83	1.80	185.40	10.50	0.01
SCR_Peaks	Performance	0.03	1.00	103.00	14.80	0.00
	SRL Phase	23.18***	1.50	154.50	9.25	0.18
	Interaction	0.05	1.50	154.50	9.25	0.00

*Note*: **p* < 0.05, ***p* < 0.01, ****p* < 0.001.
